# Splenic infarction complicating percutaneous transluminal coeliac artery stenting for chronic mesenteric ischaemia: a case report

**DOI:** 10.1186/1752-1947-2-261

**Published:** 2008-08-06

**Authors:** John A Almeida, Stephen M Riordan

**Affiliations:** 1 Gastrointestinal and Liver Unit, The Prince of Wales Hospital and University of New South Wales, Barker Street, Randwick 2031, New South Wales, Australia

## Abstract

**Introduction:**

Chronic mesenteric ischaemia is an important cause of abdominal pain, especially in older patients with risk factors for vascular disease. Until recently, surgical revascularization procedures such as endarterectomy and aorto-coeliac or aorto-mesenteric bypass grafting were the only available treatment options for patients with chronic mesenteric ischaemia. Percutaneous angioplasty and stenting have recently been shown to be effective and safe alternatives to surgical revascularization in high-risk patients with chronic mesenteric ischaemia.

**Case Presentation:**

We report an 84-year-old woman with symptoms of chronic mesenteric ischaemia, including post-prandial abdominal pain and weight loss. Investigations demonstrated calcific stenoses at the origins of the celiac, superior mesenteric and inferior mesenteric arteries, along with nonocclusive calcification in the mid-splenic artery. Coeliac artery angioplasty and stenting was performed, resulting in excellent arterial dilatation at the stenotic point and distal filling of the coeliac and superior mesenteric arteries and their branches. Within hours of successful stenting of the coeliac artery, the patient developed severe left upper quadrant pain. Progress imaging demonstrated splenic infarction, likely as a result of calcific emboli dislodged from the calcified plaque at the origin of the celiac artery at the time of angioplasty and stenting. The left upper quadrant pain resolved after 8 days and the patient remains asymptomatic 2 years post-procedure.

**Conclusion:**

This is the first reported case of splenic infarction complicating otherwise successful coeliac artery stenting, presumably as a consequence of distal embolization of disrupted calcific plaque. This complication, occurring on a background of non-occlusive splenic arterial calcification, represents a novel cause of abdominal pain post-procedure.

## Introduction

Chronic mesenteric ischaemia is an important cause of abdominal pain, especially in older patients with risk factors for vascular disease [1]. Percutaneous angioplasty and stenting have recently been shown to be effective and safe alternatives to surgical revascularization and are increasingly used in high-risk patients with chronic mesenteric ischaemia [2].

## Case presentation

**Figure 1:**
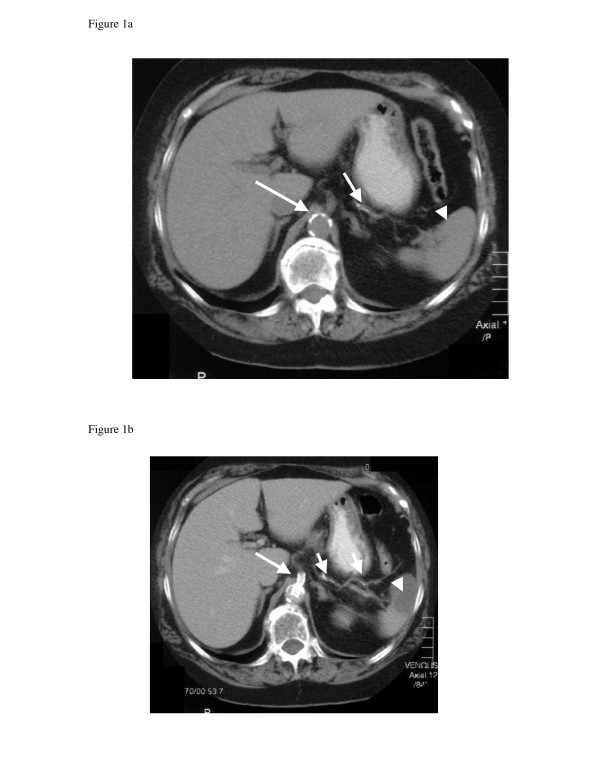
**CT scans of the abdomen**. (a) CT scan of the abdomen performed prior to percutaneous transluminal stenting of the coeliac artery, demonstrating calcified plaque in the abdominal aorta (long arrow) and mid-splenic artery (short arrow). The spleen is normal (arrowhead). (b) A progress CT scan performed 18 hours post-procedure demonstrating two wedge-shaped splenic infarcts, one of which is depicted on this view by the arrowhead. Splenic artery calcification is evident not only as before but also more distally (short arrows), in keeping with embolism of calcified plaque during coeliac artery stenting. The coeliac artery stent is pictured protruding into the abdominal aorta (long arrow).

An 84-year-old-woman with hypertension, hypercholesterolaemia and type 2 diabetes presented with symptoms of post-prandial abdominal pain and weight loss in excess of 8 kg, from 63.5 to 55 kg, over the previous 6 months. Physical examination revealed evidence of peripheral and cerebrovascular disease, with poor peripheral pulses and bruits audible over both carotid arteries. No abdominal bruits or other signs, such as abdominal tenderness, palpable masses or enlarged viscera, were evident. Faecal occult blood testing performed on three occasions was negative. Precordial examination was unremarkable. The full blood count and differential were normal. The serum albumin level was 29 g/l (normal 34 to 45 g/l). Liver biochemistry was otherwise within normal limits. The prothrombin time was similarly normal. A computed tomography (CT) scan of the abdomen showed calcified plaque in the abdominal aorta, along with nonocclusive calcification in the mid-splenic artery (Figure 1a). No abnormalities of the hollow or solid viscera were evident. Doppler studies revealed increased velocity at the origins of the coeliac, superior mesenteric and inferior mesenteric arteries consistent with significant ostial stenoses. A CT angiogram with special views of the coeliac, superior mesenteric and inferior mesenteric arteries demonstrated tight, short-segment stenoses at the origins of each of these arteries, with calcified plaque at the origins. Nonocclusive calcification in the mid-splenic artery was evident, as before. An electrocardiogram confirmed sinus rhythm. Echocardiography demonstrated no abnormality.

A diagnosis of chronic mesenteric ischaemia was made and coeliac artery stenting was performed 72 hours later, using a right brachial approach. The intention was to later stent the superior mesenteric artery as well, should flow in this artery arising from collaterals from a revascularized coeliac artery not be evident. The coeliac artery was catheterized selectively using a 6 Fr catheter, and an 8 × 26 mm balloon-mounted stent (Biotronik, Berlin, Germany) was deployed, resulting in excellent arterial dilatation at the stenotic point and distal filling of the coeliac and superior mesenteric arteries and their branches. Within several hours of successful stenting of the coeliac artery, the patient developed severe left upper quadrant pain requiring narcotic analgesia. A repeat CT scan of the abdomen performed 18 hours post-procedure revealed two wedge-shaped areas of low density within the periphery of the spleen indicative of splenic infarcts, likely as a result of calcific emboli dislodged from the calcified plaque at the origin of the coeliac artery at the time of angioplasty and stenting (Figure 1b). The vascular stent was sited within the proximal coeliac trunk. The coeliac artery distal to the stent and the superior mesenteric artery each opacified with no obvious filling defects, the latter as a result of filling via collaterals arising from the coeliac artery, demonstrating successful restoration of mesenteric blood flow and obviating the need for additional stenting of the superior mesenteric artery. There was no evidence of arterial embolism elsewhere. The patient had remained in sinus rhythm and there were no signs of infective endocarditis. The left upper quadrant pain resolved after 8 days. The splenic infarcts were no longer evident on progress CT scan performed 3 months later. The patient has experienced no further symptoms of mesenteric ischaemia now 2 years post-procedure, with a progress Doppler study demonstrating ongoing stent patency.

## Discussion

Chronic mesenteric ischaemia is an important cause of abdominal pain, especially in older patients with risk factors for vascular disease. Until recently, surgical revascularization procedures such as endarterectomy and aorto-coeliac or aorto-mesenteric bypass grafting were the only available treatment options for patients with chronic mesenteric ischaemia [1]. However, reported rates of perioperative major complications (15% to 33%) and mortality (up to 17%) are high, influenced by a high prevalence of significant patient comorbidities [2-4]. Percutaneous angioplasty and stenting have been shown to be effective and safe alternatives to surgical revascularization in high-risk patients with chronic intestinal ischaemia [2,5]. This is the first reported case of splenic infarction complicating otherwise successful coeliac artery stenting, presumably as a consequence of distal embolization of disrupted calcific plaque, with this complication occurring on a background of nonocclusive splenic arterial calcification and representing a novel cause of abdominal pain post-procedure.

## Conclusion

While coeliac artery stenting may be an effective procedure for the relief of chronic intestinal ischaemia, the possibility of complications related to distal embolism of disrupted calcific plaque should be considered, leading in this particular instance to splenic infarction.

## Abbreviations

CT: Computed tomography.

## Competing interests

The authors declare that they have no competing interests.

## Authors' contributions

Both authors contributed to the patient care and the drafting of this case report.

## Consent

Written informed consent was obtained from the patient for publication of this case report and any images. A copy of the written consent is available for review by the Editor-in-Chief of this journal.
